# Response to *Tinospora cordifolia* (giloy)‐induced liver injury during the COVID‐19 pandemic—Multicenter nationwide study from India

**DOI:** 10.1002/hep4.1989

**Published:** 2022-05-09

**Authors:** Harshad Devarbhavi

**Affiliations:** ^1^ Department of Gastroenterology St. John's Medical College Hospital Bangalore India

To the Editor,

I read with interest the article Tinospora cordifolia (Giloy)‐Induced Liver Injury During the COVID‐19 Pandemic—Multicenter Nationwide Study From India, by Kulkarni and colleagues.^[^ ^1^ ^]^ The study builds on the evidence that *T. cordifolia* causes drug‐induced liver injury (DILI) and in some cases is associated with autoantibody production and probable drug‐induced autoimmune hepatitis (DI‐AIH).^[^ ^2^ ^]^ The authors should be complimented for their detailed analysis of their patients.

The authors report the presence of autoantibodies in 60% of 43 cases, yet a diagnosis of probable DI‐AIH was established only in 18% (four of 22 patients with liver biopsy) based on the International Autoimmune Hepatitis Group's revised original score. This score is meant for research purposes and may not be ideally suited for DI‐AIH given the negative points allocated to those with exposure to hepatotoxic drugs. The simplified criteria for diagnosis of AIH was established for clinical cases^[^ ^3^ ^]^ and is preferred by experts in DILI. Liver biopsy is one of the elements in the simplified scoring system required for a “definite” diagnosis of DI‐AIH, so it will be interesting to know what proportion of patients in the Kulkarni et al. series fulfilled scores of >6 (probable) in those without histology and what proportion scored ≥7 (definite) on the Simplified Diagnostic Criteria.^[^ ^3^ ^]^ This is important, because the field of DI‐AIH is muddled with information from case reports or series where patients fulfill only partial criteria for a diagnosis of DI‐AIH (such as presence of antinuclear antibody), including a lack of liver biopsy and a range of common immunological blood tests or levels of immunoglobulin G.

Furthermore, presence of autoantibodies is not specific for autoimmune disease; antinuclear antibodies are seen in 21% of alcohol‐related liver disease and 34% of nonalcoholic fatty liver disease^[^ ^4^ ^]^ and was noted in a dilution of 1:40 in 35% of the normal population.^[^ ^5^ ^]^

The distinctive feature that separates DI‐AIH from classical AIH is that of absence of relapse in patients with DI‐AIH or progression to cirrhosis, whereas with classical AIH, relapse is almost universal. In Kulkarni and colleague's series, details of whether or not and how many were treated with steroids or immunosuppression are lacking. This information is essential, along with how many achieved remission following corticosteroid/immunosuppression withdrawal, thus qualifying for a bona fide diagnosis of DI‐AIH. The proportion that relapsed following discontinuation and/or treatment with steroids would point toward giloy acting as a trigger of *de novo* AIH in a setting of underlying liver disease, not DI‐AIH.

Increasing reports of *T. cordifolia*‐containing medications add to the growing list of newer medicinal agents, such as infliximab, adalimumab, and etanercept, but more importantly highlight the important and surreptitious role of botanical agents in causing DILI in general and DI‐AIH in particular.

## CONFLICT OF INTEREST

Nothing to report.
